# Supplementary material for: Between-module functional connectivity of the salient ventral attention network and dorsal attention network is associated with motor inhibition
Source: PLoS One. 2020 Dec 3;15(12):e0242985. doi: 10.1371/journal.pone.0242985 (PMC7714245; doi:10.1371/journal.pone.0242985)
Supplement: S1 Table — [(1) Network: Cont: control network; Default: default mode network; DorsAttn: dorsal attention network; Limbic: limbic network; SalVentAttn: salience ventral attention network; SomMot: somatomotor network; VisCent: visual central network; VisPeri: visual peripheral; (2) AAL, automated anatomical labeling atlas]. (DOCX) [file pone.0242985.s001.docx]

| Network | Hemisphere | AAL-label of nodes | X | Y | Z |
| --- | --- | --- | --- | --- | --- |
| ContA | Left | Temporal_Mid_L | -56 | -62 | 0 |
|  |  | Frontal_Mid_L | -42 | 38 | 22 |
|  |  | Frontal_Inf_Tri_L | -48 | 36 | 10 |
|  |  | Precentral_L | -40 | 8 | 34 |
|  |  | Frontal_Inf_Tri_L | -44 | 20 | 26 |
|  |  | Precentral_L | -50 | 6 | 26 |
|  |  | Frontal_Sup_L | -22 | 6 | 64 |
|  |  | Parietal_Inf_L | -34 | -46 | 40 |
|  |  | Parietal_Inf_L | -44 | -42 | 46 |
|  |  | Parietal_Sup_L | -34 | -62 | 48 |
|  |  | Parietal_Inf_L | -58 | -42 | 46 |
|  |  | Occipital_Sup_L | -28 | -74 | 42 |
|  |  | Cingulum_Ant_L | -4 | 6 | 28 |
|  | Right | Frontal_Inf_Oper_R | 38 | 10 | 34 |
|  |  | Frontal_Inf_Oper_R | 48 | 8 | 24 |
|  |  | Frontal_Inf_Tri_R | 48 | 28 | 28 |
|  |  | Frontal_Inf_Tri_R | 48 | 18 | 24 |
|  |  | Frontal_Inf_Tri_R | 50 | 30 | 18 |
|  |  | Frontal_Sup_R | 24 | 10 | 58 |
|  |  | Parietal_Inf_R | 36 | -44 | 44 |
|  |  | Parietal_Inf_R | 46 | -44 | 46 |
|  |  | Parietal_Inf_R | 54 | -32 | 52 |
|  |  | Angular_R | 36 | -70 | 46 |
|  |  | Cingulum_Mid_R | 4 | 2 | 30 |
| ContB | Left | Temporal_Mid_L | -60 | -48 | -10 |
|  |  | Temporal_Inf_L | -60 | -36 | -18 |
|  |  | Frontal_Sup_Medial_L | -4 | 28 | 48 |
|  |  | Frontal_Mid_L | -28 | 56 | 12 |
|  |  | Frontal_Sup_Orb_L | -28 | 58 | 0 |
|  |  | Frontal_Mid_Orb_L | -42 | 48 | -6 |
|  |  | Frontal_Mid_L | -30 | 14 | 58 |
|  |  | Parietal_Inf_L | -42 | -52 | 48 |
|  |  | Parietal_Inf_L | -54 | -50 | 44 |
|  |  | Angular_L | -48 | -60 | 46 |
|  | Right | Temporal_Inf_R | 64 | -42 | -10 |
|  |  | Temporal_Inf_R | 62 | -28 | -20 |
|  |  | Frontal_Sup_Medial_R | 4 | 28 | 48 |
|  |  | Frontal_Mid_R | 28 | 60 | 2 |
|  |  | Frontal_Mid_Orb_R | 42 | 50 | -6 |
|  |  | Frontal_Mid_Orb_R | 28 | 54 | -14 |
|  |  | Frontal_Mid_Orb_R | 36 | 38 | -14 |
|  |  | Frontal_Mid_R | 34 | 16 | 56 |
|  |  | Precentral_R | 42 | 6 | 50 |
|  |  | Frontal_Mid_R | 44 | 18 | 44 |
|  |  | Frontal_Mid_R | 38 | 34 | 38 |
|  |  | Parietal_Inf_R | 42 | -56 | 48 |
|  |  | Parietal_Inf_R | 56 | -40 | 48 |
|  |  | Parietal_Inf_R | 54 | -52 | 44 |
|  |  | SupraMarginal_R | 56 | -46 | 32 |
| ContC | Left | Precuneus_L | -4 | -64 | 52 |
|  |  | Precuneus_L | -10 | -78 | 46 |
|  |  | Cuneus_L | -10 | -70 | 32 |
|  |  | Cingulum_Mid_L | -4 | -22 | 30 |
|  |  | Cingulum_Post_L | -6 | -40 | 24 |
|  | Right | Precuneus_R | 8 | -72 | 52 |
|  |  | Precuneus_R | 8 | -50 | 44 |
|  |  | Precuneus_R | 6 | -64 | 44 |
|  |  | Cuneus_R | 14 | -72 | 40 |
|  |  | Precuneus_R | 16 | -64 | 28 |
|  |  | Cingulum_Mid_R | 6 | -28 | 28 |
|  |  | Precuneus_R | 8 | -44 | 20 |
| DefaultA | Left | Cingulum_Ant_L | -6 | 34 | 20 |
|  |  | Frontal_Sup_L | -16 | 68 | 8 |
|  |  | Cingulum_Ant_L | -6 | 44 | 6 |
|  |  | Frontal_Sup_Medial_L | -6 | 60 | 6 |
|  |  | Cingulum_Ant_L | -6 | 36 | -8 |
|  |  | Frontal_Med_Orb_L | -4 | 56 | -10 |
|  |  | Frontal_Mid_L | -22 | 20 | 52 |
|  |  | Frontal_Sup_L | -18 | 36 | 48 |
|  |  | Frontal_Mid_L | -24 | 28 | 44 |
|  |  | Precuneus_L | -6 | -50 | 42 |
|  |  | Precuneus_L | -2 | -68 | 42 |
|  |  | Cingulum_Mid_L | -2 | -16 | 38 |
|  |  | Cingulum_Mid_L | -4 | -34 | 38 |
|  |  | Cingulum_Post_L | -8 | -44 | 32 |
|  |  | Precuneus_L | -6 | -60 | 30 |
|  |  | Precuneus_L | -4 | -54 | 20 |
|  |  | Angular_L | -42 | -72 | 44 |
|  |  | Angular_L | -48 | -64 | 32 |
|  | Right | Temporal_Mid_R | 60 | -8 | -24 |
|  |  | Cingulum_Ant_R | 6 | 26 | 18 |
|  |  | Frontal_Sup_R | 18 | 64 | 16 |
|  |  | Cingulum_Ant_R | 8 | 54 | 12 |
|  |  | Cingulum_Ant_R | 8 | 42 | 4 |
|  |  | Frontal_Med_Orb_R | 10 | 66 | 0 |
|  |  | Frontal_Med_Orb_R | 6 | 42 | -10 |
|  |  | Frontal_Sup_R | 24 | 26 | 50 |
|  |  | Frontal_Mid_R | 26 | 34 | 38 |
|  |  | Precuneus_R | 10 | -52 | 36 |
|  |  | Cingulum_Mid_R | 4 | -20 | 36 |
|  |  | Cingulum_Mid_R | 6 | -38 | 34 |
|  |  | Precuneus_R | 4 | -64 | 32 |
|  |  | Precuneus_R | 6 | -52 | 24 |
|  |  | Angular_R | 48 | -64 | 42 |
|  |  | Angular_R | 54 | -54 | 26 |
| DefaultB | Left | Temporal_Mid_L | -52 | -22 | -6 |
|  |  | Temporal_Mid_L | -60 | -34 | -4 |
|  |  | Temporal_Mid_L | -56 | -8 | -14 |
|  |  | Temporal_Mid_L | -62 | -18 | -20 |
|  |  | Temporal_Inf_L | -54 | -2 | -30 |
|  |  | Temporal_Pole_Mid_L | -44 | 12 | -34 |
|  |  | Frontal_Inf_Tri_L | -54 | 20 | 12 |
|  |  | Frontal_Inf_Tri_L | -48 | 28 | 0 |
|  |  | Frontal_Inf_Orb_L | -46 | 32 | -10 |
|  |  | Frontal_Inf_Orb_L | -36 | 36 | -12 |
|  |  | Frontal_Inf_Orb_L | -36 | 22 | -16 |
|  |  | Precentral_L | -42 | 8 | 48 |
|  |  | Frontal_Mid_L | -40 | 20 | 48 |
|  |  | Supp_Motor_Area_L | -6 | 10 | 64 |
|  |  | Supp_Motor_Area_L | -12 | 24 | 60 |
|  |  | Frontal_Sup_Medial_L | -8 | 42 | 52 |
|  |  | Frontal_Mid_L | -22 | 50 | 32 |
|  |  | Frontal_Sup_L | -14 | 58 | 30 |
|  |  | Frontal_Sup_Medial_L | -4 | 52 | 28 |
|  |  | Angular_L | -56 | -54 | 30 |
|  |  | Temporal_Mid_L | -46 | -58 | 20 |
|  | Right | Temporal_Mid_R | 64 | -38 | 0 |
|  |  | Temporal_Mid_R | 64 | -24 | -8 |
|  |  | Frontal_Inf_Tri_R | 54 | 24 | 6 |
|  |  | Frontal_Inf_Orb_R | 48 | 32 | -8 |
|  |  | Insula_R | 34 | 22 | -18 |
|  |  | Supp_Motor_Area_R | 12 | 20 | 62 |
|  |  | Frontal_Sup_R | 14 | 38 | 52 |
|  |  | Frontal_Sup_Medial_R | 4 | 44 | 40 |
|  |  | Frontal_Sup_R | 16 | 52 | 36 |
|  |  | Frontal_Sup_Medial_R | 6 | 58 | 30 |
|  |  | Temporal_Pole_Mid_R | 50 | 8 | -32 |
| DefaultC | Left | Calcarine_L | -14 | -60 | 18 |
|  |  | Precuneus_L | -8 | -52 | 10 |
|  |  | Lingual_L | -14 | -48 | 4 |
|  |  | Fusiform_L | -18 | -38 | -12 |
|  |  | Fusiform_L | -30 | -32 | -18 |
|  |  | ParaHippocampal_L | -20 | -20 | -26 |
|  |  | Occipital_Mid_L | -40 | -78 | 30 |
|  | Right | Calcarine_R | 12 | -56 | 16 |
|  |  | Lingual_R | 14 | -46 | 4 |
|  |  | Fusiform_R | 30 | -30 | -18 |
|  |  | ParaHippocampal_R | 22 | -18 | -28 |
|  |  | Occipital_Mid_R | 46 | -76 | 30 |
|  |  | Temporal_Mid_R | 48 | -64 | 22 |
| TempPar  (DefaultD) | Left | Temporal_Mid_L | -58 | -48 | 16 |
|  |  | Temporal_Mid_L | -58 | -54 | 10 |
|  |  | Temporal_Mid_L | -52 | -44 | 4 |
|  |  | Temporal_Mid_L | -62 | -32 | 6 |
|  |  | Temporal_Mid_L | -60 | -12 | -2 |
|  |  | Temporal_Sup_L | -52 | 6 | -12 |
|  | Right | Temporal_Sup_R | 54 | -46 | 20 |
|  |  | Temporal_Sup_R | 64 | -34 | 10 |
|  |  | Temporal_Sup_R | 52 | -40 | 12 |
|  |  | Temporal_Mid_R | 60 | -46 | 6 |
|  |  | Temporal_Mid_R | 50 | -34 | 2 |
|  |  | Temporal_Sup_R | 62 | -18 | 0 |
|  |  | Temporal_Mid_R | 48 | -20 | -8 |
|  |  | Temporal_Mid_R | 54 | -4 | -14 |
|  |  | Temporal_Sup_R | 62 | -40 | 22 |
|  |  | Temporal_Pole_Sup_R | 48 | 16 | -20 |
| DorsAttnA | Left | Occipital_Inf_L | -46 | -70 | -8 |
|  |  | Temporal_Inf_L | -48 | -56 | -16 |
|  |  | Fusiform_L | -32 | -42 | -20 |
|  |  | Fusiform_L | -44 | -42 | -22 |
|  |  | Parietal_Sup_L | -28 | -62 | 62 |
|  |  | Precuneus_L | -14 | -70 | 58 |
|  |  | Parietal_Inf_L | -36 | -52 | 56 |
|  |  | Parietal_Sup_L | -28 | -58 | 50 |
|  |  | Parietal_Sup_L | -22 | -66 | 46 |
|  |  | Occipital_Sup_L | -22 | -78 | 44 |
|  |  | Occipital_Mid_L | -26 | -70 | 30 |
|  |  | Occipital_Mid_L | -32 | -84 | 26 |
|  |  | Temporal_Mid_L | -48 | -66 | 16 |
|  | Right | Temporal_Inf_R | 50 | -64 | -10 |
|  |  | Temporal_Inf_R | 50 | -50 | -18 |
|  |  | Fusiform_R | 34 | -36 | -24 |
|  |  | Parietal_Sup_R | 14 | -64 | 64 |
|  |  | Parietal_Sup_R | 26 | -58 | 60 |
|  |  | Parietal_Inf_R | 34 | -50 | 54 |
|  |  | Parietal_Sup_R | 20 | -68 | 52 |
|  |  | Parietal_Sup_R | 30 | -64 | 52 |
|  |  | Parietal_Sup_R | 18 | -78 | 50 |
|  |  | Occipital_Mid_R | 32 | -66 | 36 |
|  |  | Occipital_Mid_R | 30 | -78 | 36 |
|  |  | Occipital_Mid_R | 36 | -78 | 24 |
|  |  | Temporal_Mid_R | 54 | -56 | 12 |
|  |  | Temporal_Mid_R | 48 | -66 | 4 |
| DorsAttnB | Left | Precentral_L | -50 | 2 | 38 |
|  |  | Precuneus_L | -14 | -50 | 72 |
|  |  | Parietal_Sup_L | -20 | -56 | 66 |
|  |  | Precuneus_L | -8 | -58 | 64 |
|  |  | Parietal_Sup_L | -30 | -46 | 62 |
|  |  | Parietal_Inf_L | -38 | -38 | 50 |
|  |  | Parietal_Inf_L | -46 | -30 | 44 |
|  |  | Parietal_Inf_L | -54 | -32 | 44 |
|  |  | Parietal_Inf_L | -54 | -20 | 40 |
|  |  | SupraMarginal_L | -62 | -24 | 32 |
|  |  | Precentral_L | -30 | -8 | 52 |
|  |  | Frontal_Mid_L | -26 | 0 | 56 |
|  |  | Precentral_L | -40 | -2 | 52 |
|  | Right | Temporal_Mid_R | 60 | -54 | -2 |
|  |  | Postcentral_R | 16 | -46 | 74 |
|  |  | Parietal_Sup_R | 24 | -50 | 68 |
|  |  | Precuneus_R | 8 | -54 | 60 |
|  |  | Postcentral_R | 36 | -36 | 52 |
|  |  | Postcentral_R | 46 | -28 | 42 |
|  |  | Parietal_Inf_R | 44 | -38 | 50 |
|  |  | SupraMarginal_R | 58 | -22 | 44 |
|  |  | Postcentral_R | 62 | -14 | 30 |
|  |  | Frontal_Sup_R | 24 | -2 | 64 |
|  |  | Precentral_R | 28 | -4 | 52 |
|  |  | Precentral_R | 40 | -4 | 52 |
| LimbicA | Left | Temporal_Pole_Sup_L | -44 | 6 | -16 |
|  |  | Temporal_Pole_Sup_L | -32 | 12 | -30 |
|  |  | Temporal_Inf_L | -40 | -22 | -26 |
|  |  | Temporal_Inf_L | -54 | -22 | -30 |
|  |  | ParaHippocampal_L | -26 | -10 | -32 |
|  |  | Fusiform_L | -24 | 6 | -40 |
|  |  | Temporal_Inf_L | -38 | -6 | -42 |
|  | Right | Temporal_Inf_R | 50 | -28 | -26 |
|  |  | ParaHippocampal_R | 28 | 12 | -30 |
|  |  | Fusiform_R | 40 | -14 | -32 |
|  |  | Temporal_Pole_Mid_R | 36 | 18 | -38 |
|  |  | Temporal_Inf_R | 48 | -6 | -40 |
|  |  | Fusiform_R | 28 | -2 | -40 |
| LimbicB | Left | Frontal_Sup_Orb_L | -16 | 64 | -8 |
|  |  | Rectus_L | -4 | 24 | -20 |
|  |  | Frontal_Sup_Orb_L | -10 | 48 | -22 |
|  |  | Frontal_Inf_Orb_L | -24 | 22 | -20 |
|  |  | Frontal_Sup_Orb_L | -12 | 24 | -20 |
|  | Right | Frontal_Med_Orb_R | 10 | 62 | -14 |
|  |  | Rectus_R | 4 | 22 | -20 |
|  |  | Frontal_Mid_Orb_R | 20 | 42 | -18 |
|  |  | Rectus_R | 8 | 46 | -24 |
|  |  | Frontal_Inf_Orb_R | 22 | 22 | -20 |
|  |  | Frontal_Sup_Orb_R | 14 | 24 | -20 |
| SalVentAttnA | Left | SupraMarginal_L | -62 | -36 | 34 |
|  |  | SupraMarginal_L | -58 | -44 | 28 |
|  |  | Temporal_Sup_L | -54 | -32 | 22 |
|  |  | Precuneus_L | -6 | -48 | 56 |
|  |  | Precuneus_L | -12 | -42 | 48 |
|  |  | Cingulum_Mid_L | -12 | -28 | 42 |
|  |  | Insula_L | -36 | 4 | 10 |
|  |  | Insula_L | -32 | 18 | 8 |
|  |  | Insula_L | -40 | -14 | -2 |
|  |  | Insula_L | -38 | 2 | -4 |
|  |  | Frontal_Inf_Oper_L | -52 | 8 | 14 |
|  |  | Rolandic_Oper_L | -50 | 2 | 4 |
|  |  | Supp_Motor_Area_L | -8 | -2 | 70 |
|  |  | Supp_Motor_Area_L | -6 | 10 | 48 |
|  |  | Cingulum_Mid_L | -6 | 0 | 40 |
|  | Right | Precentral_R | 52 | 4 | 40 |
|  |  | SupraMarginal_R | 62 | -26 | 38 |
|  |  | SupraMarginal_R | 60 | -22 | 22 |
|  |  | Temporal_Sup_R | 58 | -32 | 24 |
|  |  | Paracentral_Lobule_R | 10 | -32 | 50 |
|  |  | Precuneus_R | 10 | -44 | 54 |
|  |  | Cingulum_Mid_R | 12 | -34 | 42 |
|  |  | Cingulum_Mid_R | 12 | -18 | 42 |
|  |  | Insula_R | 40 | -2 | 6 |
|  |  | Insula_R | 40 | -10 | -4 |
|  |  | Insula_R | 40 | 8 | -2 |
|  |  | Temporal_Pole_Sup_R | 40 | 6 | -16 |
|  |  | Frontal_Inf_Oper_R | 54 | 12 | 12 |
|  |  | Insula_R | 48 | 4 | 4 |
|  |  | Insula_R | 38 | 8 | 10 |
|  |  | Frontal_Sup_R | 16 | 6 | 70 |
|  |  | Supp_Motor_Area_R | 6 | -2 | 66 |
|  |  | Supp_Motor_Area_R | 6 | 10 | 58 |
|  |  | Cingulum_Mid_R | 8 | 2 | 42 |
| SalVentAttnB | Left | Cingulum_Ant_L | -6 | 22 | 32 |
|  |  | Frontal_Mid_L | -36 | 32 | 38 |
|  |  | Frontal_Mid_L | -28 | 44 | 30 |
|  |  | Frontal_Mid_L | -38 | 50 | 10 |
|  |  | Frontal_Mid_Orb_L | -26 | 50 | -14 |
|  |  | Insula_L | -44 | 12 | 2 |
|  |  | Insula_L | -32 | 24 | 0 |
|  |  | Insula_L | -34 | 16 | -8 |
|  | Right | Cingulum_Mid_R | 8 | 18 | 36 |
|  |  | Cingulum_Ant_R | 8 | 34 | 24 |
|  |  | Frontal_Inf_Tri_R | 50 | 40 | 6 |
|  |  | Frontal_Mid_R | 32 | 44 | 28 |
|  |  | Frontal_Mid_R | 26 | 54 | 24 |
|  |  | Frontal_Mid_R | 42 | 46 | 14 |
|  |  | SupraMarginal_R | 62 | -38 | 36 |
|  |  | Insula_R | 36 | 22 | 4 |
|  |  | Insula_R | 34 | 22 | -8 |
| SomMotA | Left | Precentral_L | -36 | -20 | 64 |
|  |  | Supp_Motor_Area_L | -4 | -8 | 60 |
|  |  | Paracentral_Lobule_L | -4 | -24 | 56 |
|  |  | Cingulum_Mid_L | -8 | -38 | 54 |
|  |  | Postcentral_L | -38 | -24 | 52 |
|  |  | Postcentral_L | -48 | -30 | 58 |
|  |  | Postcentral_L | -48 | -18 | 54 |
|  |  | Postcentral_L | -40 | -14 | 48 |
|  |  | Paracentral_Lobule_L | -12 | -26 | 74 |
|  |  | Postcentral_L | -18 | -40 | 72 |
|  |  | Paracentral_Lobule_L | -18 | -32 | 68 |
|  |  | Precentral_L | -14 | -12 | 72 |
|  |  | Paracentral_Lobule_L | -4 | -26 | 68 |
|  |  | Precuneus_L | -8 | -42 | 70 |
|  |  | Precentral_L | -20 | -24 | 66 |
|  |  | Precentral_L | -24 | -10 | 64 |
|  |  | Postcentral_L | -30 | -38 | 66 |
|  |  | Precentral_L | -32 | -30 | 62 |
|  |  | Cingulum_Mid_L | -8 | -16 | 48 |
|  | Right | Postcentral_R | 30 | -40 | 64 |
|  |  | Postcentral_R | 32 | -34 | 64 |
|  |  | Precentral_R | 38 | -20 | 64 |
|  |  | Postcentral_R | 44 | -22 | 54 |
|  |  | Cingulum_Mid_R | 6 | -10 | 52 |
|  |  | Postcentral_R | 50 | -26 | 56 |
|  |  | Precentral_R | 44 | -10 | 48 |
|  |  | Postcentral_R | 12 | -32 | 76 |
|  |  | Precentral_R | 52 | -12 | 50 |
|  |  | Precentral_R | 16 | -18 | 74 |
|  |  | Paracentral_Lobule_R | 6 | -22 | 72 |
|  |  | Supp_Motor_Area_R | 16 | -6 | 70 |
|  |  | Postcentral_R | 22 | -34 | 70 |
|  |  | Precentral_R | 22 | -28 | 68 |
|  |  | Precentral_R | 22 | -24 | 66 |
|  |  | Paracentral_Lobule_R | 10 | -40 | 68 |
|  |  | Frontal_Sup_R | 28 | -10 | 64 |
|  |  | Supp_Motor_Area_R | 4 | -24 | 58 |
|  |  | Postcentral_R | 34 | -28 | 62 |
|  |  | Postcentral_R | 54 | -16 | 40 |
| SomMotB | Left | Postcentral_L | -62 | -18 | 20 |
|  |  | Rolandic_Oper_L | -48 | -24 | 18 |
|  |  | Rolandic_Oper_L | -60 | -2 | 10 |
|  |  | Rolandic_Oper_L | -48 | -12 | 14 |
|  |  | Rolandic_Oper_L | -36 | -26 | 18 |
|  |  | Insula_L | -38 | -8 | 12 |
|  |  | Heschl_L | -36 | -24 | 10 |
|  |  | Postcentral_L | -48 | -16 | 40 |
|  |  | Postcentral_L | -52 | -6 | 44 |
|  |  | Postcentral_L | -54 | -8 | 30 |
|  |  | Postcentral_L | -62 | -10 | 32 |
|  |  | Postcentral_L | -60 | -2 | 24 |
|  |  | Temporal_Sup_L | -40 | -36 | 14 |
|  |  | Temporal_Sup_L | -58 | -36 | 16 |
|  |  | Temporal_Sup_L | -56 | -22 | 8 |
|  |  | Temporal_Sup_L | -50 | -10 | 0 |
|  | Right | Postcentral_R | 62 | -12 | 16 |
|  |  | Rolandic_Oper_R | 48 | -22 | 18 |
|  |  | Rolandic_Oper_R | 60 | 0 | 10 |
|  |  | Rolandic_Oper_R | 50 | -10 | 14 |
|  |  | Temporal_Sup_R | 42 | -30 | 18 |
|  |  | Rolandic_Oper_R | 42 | -14 | 18 |
|  |  | Insula_R | 34 | -22 | 14 |
|  |  | Insula_R | 38 | -8 | 14 |
|  |  | Insula_R | 40 | -20 | 4 |
|  |  | Postcentral_R | 52 | -6 | 38 |
|  |  | Postcentral_R | 60 | -6 | 26 |
|  |  | Precentral_R | 60 | 6 | 30 |
|  |  | Temporal_Sup_R | 60 | -24 | 10 |
|  |  | Temporal_Sup_R | 54 | -14 | 6 |
|  |  | Temporal_Pole_Sup_R | 52 | 4 | -6 |
| VisCent | Left | Calcarine_L | -8 | -98 | -8 |
|  |  | Occipital_Mid_L | -40 | -84 | 14 |
|  |  | Occipital_Mid_L | -24 | -96 | 6 |
|  |  | Occipital_Mid_L | -46 | -74 | 6 |
|  |  | Occipital_Mid_L | -42 | -86 | -4 |
|  |  | Lingual_L | -24 | -96 | -12 |
|  |  | Lingual_L | -16 | -86 | -16 |
|  |  | Fusiform_L | -36 | -82 | -16 |
|  |  | Fusiform_L | -24 | -72 | -10 |
|  |  | Occipital_Mid_L | -26 | -84 | 22 |
|  |  | Cuneus_L | -10 | -96 | 18 |
|  |  | Fusiform_L | -36 | -62 | -16 |
|  | Right | Calcarine_R | 8 | -92 | -2 |
|  |  | Occipital_Mid_R | 44 | -78 | 10 |
|  |  | Occipital_Mid_R | 24 | -98 | 8 |
|  |  | Occipital_Mid_R | 36 | -88 | 2 |
|  |  | Occipital_Inf_R | 26 | -96 | -10 |
|  |  | Occipital_Inf_R | 42 | -84 | -12 |
|  |  | Lingual_R | 20 | -88 | -12 |
|  |  | Fusiform_R | 24 | -74 | -10 |
|  |  | Fusiform_R | 38 | -74 | -16 |
|  |  | Occipital_Mid_R | 28 | -88 | 20 |
|  |  | Cuneus_R | 12 | -94 | 18 |
|  |  | Fusiform_R | 36 | -54 | -18 |
| VisPeri | Left | Calcarine_L | -8 | -74 | 10 |
|  |  | Calcarine_L | -4 | -88 | 2 |
|  |  | Cuneus_L | -12 | -82 | 36 |
|  |  | Occipital_Sup_L | -16 | -88 | 34 |
|  |  | Cuneus_L | -12 | -72 | 20 |
|  |  | Cuneus_L | -2 | -84 | 24 |
|  |  | Calcarine_L | -18 | -64 | 6 |
|  |  | Lingual_L | -14 | -56 | 2 |
|  |  | Lingual_L | -14 | -44 | -6 |
|  |  | Lingual_L | -6 | -76 | -6 |
|  |  | Lingual_L | -12 | -62 | -4 |
|  |  | Lingual_L | -24 | -54 | -8 |
|  | Right | Calcarine_R | 22 | -60 | 6 |
|  |  | Calcarine_R | 10 | -74 | 8 |
|  |  | Occipital_Sup_R | 16 | -86 | 36 |
|  |  | Cuneus_R | 14 | -78 | 34 |
|  |  | Cuneus_R | 4 | -80 | 24 |
|  |  | Calcarine_R | 16 | -66 | 18 |
|  |  | Lingual_R | 18 | -44 | -2 |
|  |  | Lingual_R | 14 | -58 | -4 |
|  |  | Lingual_R | 10 | -72 | -6 |
|  |  | ParaHippocampal_R | 18 | -36 | -12 |
|  |  | Lingual_R | 26 | -52 | -8 |
|  |  |  |  |  |  |
