# Supplementary material for: Between-module functional connectivity of the salient ventral attention network and dorsal attention network is associated with motor inhibition
Source: PLoS One. 2020 Dec 3;15(12):e0242985. doi: 10.1371/journal.pone.0242985 (PMC7714245; doi:10.1371/journal.pone.0242985)
Supplement: Attachment — Submitted filename: Response to Reviewers.docx [file pone.0242985.s003.docx]

Reviewer #1: Overview

In this paper, Hsu and colleagues use resting-state fMRI in distributed brain networks to predict individual differences in task performance (in this case, the stop-signal task). In particular, for each of the seventeen brain networks they analyzed, they calculated two graph-theoretic statistics (Participation Coefficient, PC, which measures between network connectivity, and Within-module-degree, WMD, which measures within network connectivity) and trained a multiple linear regression model to predict SST performance across subjects. The paper’s motivations and scope are, in general, well-written and clear. However, there are serious concerns with the statistical methods that were employed. Therefore, I believe that the manuscript is not currently suited for publication in its current form. If the authors can address these all of these concerns, I would be happy to look at a revised version. The following contains my specific concerns with the current publication in order of how important I think they are.

Reply0: The reviewer’s understanding of this study is correct. Regarding the concerns about methodology employed, we have adjusted the use of statistical models and provided new results to address your concerns.

1. Problem with training paradigm of regression model:
The authors train a linear regression model to predict stop-signal task performance across subjects using graph-theoretic statistics of the functional brain networks. It seems that the authors did not evaluate the linear regression model on a set of held-out subjects. If this is the case, then the authors will not be able to claim that these network statistics can predict individual differences in behavior. The current results can only suggest that there is a potential linear association between these network statistics and the individual differences in behavior. In order to show brain data can predict individual differences in behavior for a particular dataset, one must employ some kind of cross-validation paradigm where predictions from the regression model are evaluated on subjects that were not included in the training of the regression model. This is a standard procedure for studies that use functional brain data to explain individual differences in behavior. For example, Rosenberg et al. 2016 Nature Neuroscience use leave-one-out cross validation in their internal validation analyses to demonstrate their model accurately predict behavioral scores of unseen subjects. Additionally, they also show that their models have strong generalizability by showing their linear regression models trained on a dataset with a particular behavior measure and set of subjects generalize to a dataset with a completely different set of subjects and a different behavioral measure. Generalizing to different task performance measures might be out of the scope in the current study, but I think it is reasonable to ask the authors to employ a more principled approach in terms of having test sets of held-out subjects during training.

Reply1: We thank the reviewer for this suggestion on strengthening the rationale behind the use of regression model to predict behavioral performance. We agree with the reviewer’s view to use leave-one-out cross validation for the prediction. Therefore, we have revised the manuscript accordingly.

2. Reporting p-values
I was not able to find details on how the p-values were determined for reporting the Pearson correlation that determined model performance in the current study. I understand that they were corrected for using Bonferroni corrections, but how exactly were the original uncorrected p-values determined before they were subsequently corrected? Were they determined parametrically or non-parametrically? I recommend the p-values be determined non-parametrically, because a non-parametric approach would not make any assumptions on the data and thus one would avoid possibly violating certain assumptions that invalidate the p-value. If the authors decide to employ cross-validation to address my first point above, then this point will be even more relevant. Here is an example of calculating a p-value non-parametrically (the permutation test): shuffle the participants’ task performance, perform the whole analysis pipeline, calculate regression performance (in this study’s case, Pearson’s correlation), and repeat procedure many times and report the percentage of iterations that had a Pearson’s correlation as high as the one obtained from unshuffled data. This procedure should be rigorous enough to obtain reliable p-values in the current study regardless of the analysis pipeline.

Reply2: We thank the reviewer’s suggestion. We have included non-parametrical p-values (see Table 3) for both association and prediction results as you suggested. We find out that participation coefficients (i.e., PC) of salient attention A network and dorsal attention A network show significance (salient attention A network: p = 0.02; dorsal attention A network: p = 0.04).

3. Addressing head motion during analysis stage in addition to the preprocessing stage. The effect of head motion on functional connectivity analyses is well documented. It’s good that head motion parameters were regressed out in the preprocessing stage, but I think it is important that head motion is ensured to not be affecting the main analyses. Hsu et al. 2018 Social Cognitive and Affective Neuroscience is a similar study to the current study in that the authors are also using strength of functional brain network to predict individual differences in behavior and assess their predictions by correlating predicted behavior scores from brain activity to actual behavior scores (in their case, the behaviors were personality traits). This study addressed head motion by using partial Pearson correlations between observed scores and predicted scores to control for head motion. A similar kind of control for head motion in both the preprocessing and analysis stage (rather than only in the preprocessing stage) would greatly strengthen the current work by ensuring head motion is not confounding the main results.
Reply3: We thank the reviewer’s suggestion. We have included an additional step for assessing the prediction by running the partial correlation between observed and predicted scores to control for head motion (see p.12, section “Image pre-processing”, lines 239-241).

4. Clarity on describing the brain network statistics
Although the authors point to previous work explaining how to calculate PC and WMD and include useful high-level descriptions in the current manuscript, I think it would be useful for them to include a specific section with precise mathematical descriptions of how these graph-theory statistics are calculated. Doing so will make it clearer for readers interested in reproducing the study’s results.
Reply4: We thank the reviewer’s suggestion for improving readability. As you suggested, we have incorporated more detailed descriptions on the calculation of PC and WMD in the introduction and methods sections of the manuscript.

5. Clarity on results figure.
Figure 2a contains the effect sizes (Pearson correlations) from the statistical analysis of each of the brain networks (predicting SST performance from graph-theoretic statistics). It would be useful for the figure to mark which brain networks yielded statistically significant results (possibly with a star next to the network name). Additionally, in order to have a better sense of the variance of the correlation, it would be nice if the authors included error bars to each of the bars in Figure 2a. I recommend these error bars be 95% confidence intervals calculated non-parametrically using the bootstrap method (for the same reasons I addressed in point 2 when I suggested using non-parametric p-value calculations).
Reply5: We thank the reviewer’s suggestion on figure presentation. We have included error bars in the figures throughout the manuscript. Moreover, your suggestion together with your previous comments on non-parametric p-values were also calculated and presented in the figures.

6. Performing a multiple regression across all brain networks
The authors did a regression for each individual brain network to predict subjects’ individual differences in SST performance. Why not do a multiple linear regression that uses all 17 brain networks to predict the behavior scores? This could give interesting information on how much each brain network is contributing to the prediction (using the magnitude of the regression weights that have been assigned to each of the brain networks).
Reply6: We thank the reviewer’s suggestion. We did originally consider using a multiple linear regression model across all 17 brain networks to predict individual differences in SST performance. However, it would occur a statistical problem (i.e. Multicollinearity) in which independent variables in a regression model are correlated. This correlation is a problem because independent variables should be independent. The high degree of correlations between networks would cause the coefficients not reliable to interpret/inference the results of networks’ contributions in explaining SST performance.

7. Clarity on preprocessing section
Section 2.3.2 is a little unclear to me. Nuisance covariates were regressed out in the second step, and then certain parameters are set as nuisance covariates in the third step and a filter is applied on them in the fourth step. I’m a little confused on what role these third and fourth steps are playing.
Reply7: We thank the reviewer’s suggestion. We have modified this section to clarify the preprocessing steps, especially the third and fourth steps in the section of image pre-processing (see p.12, section “Image pre-processing”, line 235-238). Combining with the third and fourth steps in the previous version, in the third step of the new version, we regressed out bad frames at the subject level detected by “head motion censoring” and [R R^2^ R_t-1_ R^2^_t-1_], where t and t-1 refer to the current and immediately preceding timepoint, and R refer to nuisance covariates.

8. Small error in describing previous work.
Line 124 indicates “there is no prior research employing graph-theoretic analysis methods on network properties of rs-fMRI data to associate with SSRT.” However, Kumar et al. 2019 Brain and Behavior, which is cited previously in the introduction as citation 24, use a graph theory quantity (maximum flow) to predict task performance in a dataset that employs the Stop Signal Task. The difference is that the current study is using different graph-theoretic quantities and focuses on motor inhibition (whereas Kumar and colleagues focus on attention) but this detail should be emphasized.
Reply8: We thank the reviewer’s suggestion. We have included a paper by Kumar et al. (2019) in this paragraph and focused on differences between their work and the current study.

9. Minor points
In line 149, I think “form” should be “from.”
Reply9: We thank reviewer’s correction. We have corrected the typo.

References
1. Rosenberg, M. D., Finn, E. S., Scheinost, D., Papademetris, X., Shen, X., Constable, R. T., & Chun, M. M. (2016). A neuromarker of sustained attention from whole-brain functional connectivity. Nature neuroscience, 19(1), 165-171.
2. Hsu, W. T., Rosenberg, M. D., Scheinost, D., Constable, R. T., & Chun, M. M. (2018). Resting-state functional connectivity predicts neuroticism and extraversion in novel individuals. Social cognitive and affective neuroscience, 13(2), 224-232.
3. Kumar, S., Yoo, K., Rosenberg, M. D., Scheinost, D., Constable, R. T., Zhang, S., ... & Chun, M. M. (2019). An information network flow approach for measuring functional connectivity and predicting behavior. Brain and behavior, 9(8), e01346.

Reply: We have added the first and second references in the method section (see p.14, line 293-295; p.15, line 295-302) and modified the study interpretation of Kumar et al. (2019) in the introduction section (see p.6, line 112-117).

Reviewer #2: In this manuscript, the authors used resting-state fMRI to investigate associations between motor inhibition and connectomics. The methods are sound, the manuscript clearly written and the conclusions supported by the data. The large sample size is a strength of this study. I have nevertheless some comments/suggestions which I strongly believe would improve the manuscript and clarify its impact.

Reply: We thank the reviewer’s positive feedbacks. We have incorporated your suggestions for improving this manuscript.

Major points:
Introduction: The rationale for a graph-theory analysis in this context needs further elaboration; while, the authors present the characteristics of this analysis and its potential to unravel topological features, it is not clear why/how this approach will bring new insights to answer this specific research question

Reply: We thank the reviewer’s suggestion about the rationale behind the use of graph-theory analysis on motor inhibition. As we reviewed in the introduction, previous works on association between functional networks and motor inhibition have shown potential functional brain involvement at network level. However, such attempts did not consider its association between functional network organization properties and performance of motor inhibition. Moreover, how the results on topological features of functional network properties associated with previous findings remain unclear. Therefore, our goal of this study is to explore whether within- or between- network connection properties associated with motor inhibition. Specifically, how do these topological features associate with motor inhibition and its relation to previous findings. These results would substantially enhance our understanding of functional networks involvement in motor inhibition.

Methods: Please add reference of ethical approval

Reply: We thank the reviewer’s suggestion. We have reported the case number of ethical approvals by the National Cheng Kung University Research Ethics Committee.

Participants: How did the authors decide on sample size? The manuscript does not contain any evidence of power calculation.

Reply: We add the power analysis result of each network property model in table 3. In the power analysis, the degree of freedoms and R-squared were extracted from each multiple linear regression model, and the alpha is set as 0.05. The desired power for the motion-controlled partial correlation models should conventionally be at least higher than 0.80. Our result showed that the power for salient attention A network and dorsal attention A network result in 0.95 and 0.90 respectively, which fit our criteria, suggesting the model of these two network properties were sufficient.

MRI: Can the authors expand on the specific instructions given to the participants for the resting-state scan? Did the authors check by any mean alertness during the scan? Were the scans acquired at the same time of the day? Further information on this extra sources of variability would be interesting to know

Reply: The participants were not limited to be scanned at the same time of the day, but they were all scanned during the day time. During the resting-state functional scans, the participants were instructed to remain awake and relax with their eyes open and stare at the white cross as shown on the screen (each scan lasted for 8min for each participant).

Preprocessing: Did the authors perform any QC on movement beyond censoring "bad volumes"? Did they exclude any subject because of excessive movement? What were the maximum number of censored volumes the authors thought to be acceptable for including a subject?

Reply: The screening criteria for imaging quality control were based on head motion parameters and frame‐wise displacement (FD). None of the remaining participants' max head motion exceeded 2.5 mm, or means FD exceeded 0.25. We also visual inspection of all images after normalization and coregistration steps to make sure there was no bad warping. The subjects with 2.5 mm and 2.5 degree in max head motion would be asked for re-scanning. No maximum numbers of censored volumes were set as criteria for excluding a subject, but volumes of the subject motion scrubbing over 0.9 mm would be set as a covariate at the preprocessing level.

Connectomics: The description of the methods for calculating the PC and WMD is highly insufficient; in the absence of this information, the reader cannot follow or scrutinize the approach taken by the authors to derive these metrics

Reply: We thank the reviewer’s suggestion for readability. To address this, we have included an additional section to describe its mathematical logics behind the PC and WMD.

Multiple regression: Were the assumptions of this statistical model verified? If yes, how? Also, given the large sample size i feel it would strength the message if the authors would consider including a further cross-validation analysis (k-folds) to examine how well the model will generalize to new observations; Given their focus on the somatomotor network, are the authors confident that this association is not explained by inter-individual differences in head movement? Did authors control for head movement (i.e. mean framewise displacement) in their regression models?

Reply: We add the head motion in the regression model and conduct the leave-one-out cross-validation.

Discussion/Conclusion: I am afraid the reader is left without a clear understanding of what this study brings that is new; please consider expanding on the contribution and implication of these findings
Reply: We thank the reviewer’s suggestion. As you suggested, we have expanded contribution and implication of current findings in this study.

Minor points:
Line 398: "shows more explanations" - please rephrase

Reply: We thank the reviewer’s correction. We have rephrased the sentence.
