# Supplementary material for: Between-module functional connectivity of the salient ventral attention network and dorsal attention network is associated with motor inhibition
Source: PLoS One. 2020 Dec 3;15(12):e0242985. doi: 10.1371/journal.pone.0242985 (PMC7714245; doi:10.1371/journal.pone.0242985)
Supplement: Attachment — Submitted filename: R2_Response to Reviewers.docx [file pone.0242985.s004.docx]

Review Comments to the Author

Reviewer #1: I believe the authors have provided a very comprehensive revision and have addressed the majority of my concerns. The paper is much stronger than before, but there are three main issues that I think need to be addressed before this paper is appropriate for publication. If these can be addressed, I think this paper will be well-suited for publication. The following are the aforementioned issues, in order of how crucial I believe they are:

1. Accounting for multiple comparisons in the determination of statistical significance

The authors have done a great job in using cross-validation to generate and evaluate predictions on held-out subjects as well as utilizing permutation testing and non-parametric p-values. However, since there are many of these statistical tests being done (seventeen brain networks and two graph-theory statistics for 34 statistical tests in total). It is absolutely necessary to adjust the statistical tests for the multiple comparisons issue. The previous iteration of the manuscript used the Bonferroni method, which is a great method to use for multiple comparisons, but it seems that this has been taken out in the current manuscript.

Providing a statistical test that incorporates both non-parametric permutation testing as well as takes into account for multiple comparisons will be the best way to ensure the study’s results are statistically solid enough for publication.

The authors could use Bonferroni in this situation, but I do recognize that Bonferroni is highly conservative and it may be that none of the authors’ results may end up being statistically significant if they use Bonferroni. I would be fine if the authors used a less conservative method of multiple comparisons, but I think it is absolutely necessary to at least incorporate some kind of statistical correction for multiple comparisons.

**Reply: We thank the reviewer for this advice. The difference in statistical analyses between the first submission and first revision is that the first manuscript reported the parametric p values of correlations between the predicted and observed SSRT, while the second manuscript reported partial correlation between predicted and observed SSRT, which partial out effects from motion parameters, and reported non-parametric permutation value. After controlling for multiple comparisons for all the 34 models tested, with more liberal methods, we did not find a corrected p value less than .05.**

**However, we still think it is worthwhile to report our findings. Therefore, we implemented Bayes Factor (BF_10_), which used thresholding methods to make their inference more conservative (Han et al. 2019). Specifically, the Bayesian procedure is more conservative, making fewer claims with confidence and not overestimating effect sizes. In this case, we used Bayes Factors for this revised version to evaluate the evidence strength of each model. The BF_10_ is the ratio of the likelihood of an alternative hypothesis (in the present case, the presence of the correlation) to the likelihood of the null hypothesis (in the present case, the absence of the correlation**)**.** **Bayesian inference based on BF_10_ would enable us evaluate the relative strength of evidence supporting the null or alternative hypothesis, which could not be easily done within the frequentist inference. Moreover, Bayesian statisticians have argued that it is relatively freer from issues associated with inflated false positives and multiple comparison correction. This is because Bayesian statistics do not rely on frequentist assumptions associated with false positives but focuses on updating the posterior probability distributions of parameters of interest based on data (**[**Gelman, Hill, & Yajima, 2012**](https://www.biorxiv.org/content/10.1101/855460v1.full#ref-8)**;** [**Han & Park, 2018**](https://www.biorxiv.org/content/10.1101/855460v1.full#ref-18)**). Furthermore, researchers have suggested that Bayes factor can also solve the issue of multiple comparisons (please see Neath & Cavanaugh, 2006 for details). For instance, BF_10_ = 4 may be interpreted as the data being 4 times more likely to occur under the alternative hypothesis than under the null hypothesis. The interpretation of BF_10_ can base on Wetzels & Wagenmakers (2012), which edited from Jeffreys (1961). They suggested that the criteria of 1 < BF_10_ < 3 can only be anecdotal evidence for the alternative hypothesis, while the criteria of 3 < BF_10_ < 10 can be interpreted as moderate evidence sufficiently supporting the alternative hypothesis (for review, please see Ly, A., et al., 2016). Therefore, the BF_10_ values of significant models in partial correlation are reported. The result finds that the PC of salient ventral attention A network (BF10 = 4.01) is sufficiently supporting the alternative hypothesis, while the PC of dorsal attention A network (BF_10_ = 1.96) is only weak and less sufficient evidence of alternative attention. We have added the description of this new result in the revised manuscript. Please refer to following pages: [p.15, line 316-325; p.17, line 345-346, p.19, line 374-376].**

**Reference**

**Gelman, A., Hill, J., & Yajima, M. (2012). Why We (Usually) Don’t Have to Worry About Multiple Comparisons. J. Res. Educ. Eff. 5 (2), 189–211. doi:10.1080/19345747.2011.618213**

**Han, H., Glenn, A. L., & Dawson, K. J. (2019). Evaluating Alternative Correction Methods for Multiple Comparison in Functional Neuroimaging Research. Brain Sci. 9 (8), 198. doi:10.3390/brainsci9080198.**

**Han, H. & Park, J. (2018). Using SPM 12’s Second-level Bayesian Inference Procedure for fMRI Analysis: Practical Guidelines for End Users. Frontiers in Neuroinformatics, 12, 1. doi:10.3389/FNINF.2018.00001**

**Jeffreys, H. (1961) Theory of Probability. 3rd Edition, Clarendon Press, Oxford.**

**Ly, A., et al. (2016). "An evaluation of alternative methods for testing hypotheses, from the perspective of Harold Jeffreys." Journal of Mathematical Psychology 72: 43-55.**

**Neath, A. A., & Cavanaugh, J. E. (2006). A Bayesian approach to the multiple comparisons problem. *Journal of Data Science*, *4*(2), 131-146.**

**Wetzels, R. and E.-J. Wagenmakers (2012). "A default Bayesian hypothesis test for correlations and partial correlations." Psychonomic Bulletin & Review 19(6): 1057-1064.**

2. Error in displaying error bars in Figure 2a.

The authors have added error bars in Figure 2a to give an estimate of the effect size of the regression models’ performance, which is a great addition. However, I think there has been a mistake in adding these error bars. The error bars are at the beginning of each bar in the barplot (where it starts at 0) instead of the end of the bar (which is where the calculated R value lies). This should be fixed immediately as it makes the figure very confusing.

**Reply: We thank the reviewer for this suggestion, and we have corrected Fig 2a according to the reviewer’s comments.**

3. Clarity in displaying which brain networks/graph-theory statistics are significant

Table 3 displays r and p-values for each of the statistical tests. It is difficult to find which one is significant, so it would be helpful for the authors to bold-face the rows in which the p-value was significant. Related to this, it is similarly difficult to find which brain network/graph theory statistic was significant in Figure 2. A similar bold-facing on the network name would be really helpful here as well.

**Reply: We thank the reviewer for this suggestion, and we modify the Table 3 and Fig 2a according to the suggestion.**

Reviewer #2:

The authors have addressed most of my previous comments. However, I am afraid that the power analyses the authors now added need some further contextualization. The authors used the degrees of freedom and effect sizes from their own data to provide a picture of actual achieved power for each regression model. This does not inform the reader on how the sample size was decided, but can provide information for appraising each individual finding. This needs to be better explained, otherwise these analyses sound rather seldom. Also, if it was the case that a priori power analyses to decide on sample size were not conducted, please state this openly - I don't see a major problem here, but for clarity it is important that the reader is aware of such circumstance.

**Reply: We thank you for this comment. We did not conduct the priori power analysis to decide on sample size, yet we are willing to clarify this circumstance openly in our research [see p.14, line 287 – 288]. In our previous revision, we have incorporated the post-hoc power analysis on each network property model in table 3. In the power analysis, the degree of freedoms and R-squared was extracted from each multiple linear regression model, and the alpha is set as 0.05. The desired power for the motion-controlled partial correlation models should conventionally be at least higher than 0.80. Our result showed that the power for salient attention A network and dorsal attention A network result in 0.95 and 0.90 respectively, which fit our criteria, suggesting the model of these two network properties was sufficient. Moreover, any power analysis question requires consideration of effect sizes, which we have detailed in the manuscript. Moreover, commonly presume that the required sample size for linear regression analysis is based on predictor variables or participant numbers (e.g. above 50 participants) or both (5 participants/ten participants for each predictor variable). Studies (Green, 1991; Jenkins & Quintana-Ascencio, 2020; Wilson Van Voorhis & Morgan, 2007) have shown that a minimum N>25 is essential (Jenkins & Quintana-Ascencio, 2020) for accurate inference but sample size ranges from N ≥ 50 + 8m (recommendation of Green (1991)) for the multiple regression or N ≥ 104 + m for partial correlation where m is the number of predictor variables. Moreover, Knofczynski & Mundfrom (2008) proposed a guideline for determining the minimum required sample size for using multiple regressions for prediction. They proposed that the sample should vary from small to large, according to the effect sizes. As different guidelines provide some useful reference for deciding sample size, the larger sample size would indeed provide more robust insight into this study. As of now, our inference based on current sample size may be still justified.**

**References:**

**Green, S. B. (1991). How Many Subjects Does It Take To Do A Regression Analysis? Multivariate Behavioral Research. https://doi.org/10.1207/s15327906mbr2603_7.**

**Jenkins, D. G., & Quintana-Ascencio, P. F. (2020). A solution to minimum sample size for regressions. PLoS ONE. https://doi.org/10.1371/journal.pone.0229345.**

**Knofczynski, G. T., & Mundfrom, D. (2008). Sample Sizes When Using Multiple Linear Regression for Prediction. Educational and Psychological Measurement, 68(3), 431–442. https://doi.org/10.1177/0013164407310131**

**Wilson Van Voorhis, C. R., & Morgan, B. L. (2007). Understanding Power and Rules of Thumb for Determining Sample Sizes. Tutorials in Quantitative Methods for Psychology. https://doi.org/10.20982/tqmp.03.2.p043**
