# Supplementary material for: Between-module functional connectivity of the salient ventral attention network and dorsal attention network is associated with motor inhibition
Source: PLoS One. 2020 Dec 3;15(12):e0242985. doi: 10.1371/journal.pone.0242985 (PMC7714245; doi:10.1371/journal.pone.0242985)
Supplement: Attachment — Submitted filename: R3_Response to Reviewers.docx [file pone.0242985.s005.docx]

Response to Editor and Reviewers’ comments

PONE-D-20-17526R2
Between-module functional connectivity of the salient ventral attention network and dorsal attention network is associated with motor inhibition
PLOS ONE

Editor’s comment:
Please address the comment from Review 1 regarding the error bars. If there is some reason that you wish to keep the Figure as-is, please provide a specific rationale.

REPLY: We have modified the figure as requested by Reviewer 1.

**Comments to the Author**
6. Review Comments to the Author

Reviewer #1: I believe the major comments on the statistics have been met, but I do request that the error bars in Figure 2 for SalVentAttnB in PC be corrected. It seems the bars aren't centered on the end of the bar. It is very important that these error bars are placed correctly, so I implore the authors to double check this figure.

REPLY: We have modified the figure as requested. Please see the revised Figure 2. Please note the bootstrapped confidence intervals are not symmetric, because the empirical null distribution is skewed.
